# Complex PTSD and phased treatment in refugees: a debate piece

**DOI:** 10.3402/ejpt.v7.28687

**Published:** 2016-02-12

**Authors:** F. Jackie June ter Heide, Trudy M. Mooren, Rolf J. Kleber

**Affiliations:** 1 Foundation Centrum'45, Oegstgeest/Diemen, The Netherlands | Partner in Arq Psychotrauma Expert Group; 2 Department of Clinical & Health Psychology, Utrecht University, The Netherlands

**Keywords:** Posttraumatic stress, trauma, torture, prevalence, efficacy, ISTSS treatment guidelines, asylum seekers, ICD-11, narrative exposure therapy, culturally adapted cognitive-behaviour therapy

## Abstract

**Background:**

Asylum seekers and refugees have been claimed to be at increased risk of developing complex posttraumatic stress disorder (complex PTSD). Consequently, it has been recommended that refugees be treated with present-centred or phased treatment rather than stand-alone trauma-focused treatment. This recommendation has contributed to a clinical practice of delaying or waiving trauma-focused treatment in refugees with PTSD.

**Objective:**

The aim of this debate piece is to defend two theses: (1) that complex trauma leads to complex PTSD in a minority of refugees only and (2) that trauma-focused treatment should be offered to all refugees who seek treatment for PTSD.

**Methods:**

The first thesis is defended by comparing data on the prevalence of complex PTSD in refugees to those in other trauma-exposed populations, using studies derived from a systematic review. The second thesis is defended using conclusions of systematic reviews and a meta-analysis of the efficacy of psychotherapeutic treatment in refugees.

**Results:**

Research shows that refugees are more likely to meet a regular PTSD diagnosis or no diagnosis than a complex PTSD diagnosis and that prevalence of complex PTSD in refugees is relatively low compared to that in survivors of childhood trauma. Effect sizes for trauma-focused treatment in refugees, especially narrative exposure therapy (NET) and culturally adapted cognitive-behaviour therapy (CA-CBT), have consistently been found to be high.

**Conclusions:**

Complex PTSD in refugees should not be assumed to be present on the basis of complex traumatic experiences but should be carefully diagnosed using a validated interview. In line with treatment guidelines for PTSD, a course of trauma-focused treatment should be offered to all refugees seeking treatment for PTSD, including asylum seekers.

Many asylum seekers and refugees have fled their country of origin to escape the horrors of war, persecution, organised violence, or torture. Based on these traumatic experiences, in the posttraumatic stress literature two claims are often made: first, that refugees are at increased risk of developing complex posttraumatic stress disorder (complex PTSD; e.g., Cloitre et al., 2009) and second, that refugees should be treated with present-centred or phased treatment rather than stand-alone trauma-focused treatment (e.g., National Institute for Clinical Excellence [NICE], 2005). Although the psychological consequences of prolonged and extreme traumatisation have been subject of research and debate for several decades (e.g., Niederland, 1971), in recent years the subject has gained a new impetus. In 2012, the *ISTSS Expert Consensus Treatment Guidelines for Complex PTSD in Adults* (Cloitre et al., 2012) were published, which address complex PTSD in refugees. Also, an official complex PTSD diagnosis has been proposed for inclusion in the 11th version of the *International Classification of Diseases* (ICD-11; Maercker et al., 2013) in 2018. In the last decade, research on refugee pathology and treatment has also been accumulating. Timing therefore seems right to debate the validity of the above claims. To this aim in this paper we formulate two theses: (1) that complex trauma leads to complex PTSD in a minority of refugees only and (2) that trauma-focused treatment should be offered to all refugees who seek treatment for PTSD. We defend these theses using the state-of-the art of research on complex PTSD and treatment in refugees. However, before we do so, we describe the central terminology used in the complex PTSD field (complex trauma, complex PTSD, and trauma-focused and phased treatment) as well as how the central assumptions of this field have been appraised.

## Terminology

Research and treatment of complex PTSD centre around the hypothesis that complex traumatic experiences (complex trauma) lead to a posttraumatic syndrome (complex PTSD) that is clearly distinguishable from regular posttraumatic stress disorder (PTSD; American Psychiatric Association, 2013). The terms “complex trauma” and “complex PTSD” are often used interchangeably, a practice which may lead to confusion. Courtois and Ford (2009) propose a clear distinction between complex trauma and complex PTSD, with complex trauma referring to complex traumatic experiences, and complex PTSD to complex posttraumatic symptoms. Although this use of terminology may be debated (e.g., Mooren & Stöfsel, 2015), in this paper we follow that distinction.

## Complex trauma

The experience of war has been a central element in the search for a distinction between relatively delineated traumatic events, such as a robbery, disaster, or traffic accident, and more complicated traumatic events. This search mainly stems from the second half of the twentieth century, when the psychological aftermath of World War II was being explored (e.g., Eitinger, 1980). In the 90s, Terr (1991) and Herman (1992a, b) broadened this search to include the experience of (domestic) violence in children and women. They suggested that a meaningful clinical distinction may be made between single traumatic events and repeated, prolonged, interpersonal traumatic events occurring in a context of totalitarian control. This clinical definition of complex trauma has since gone virtually unchanged, with the ISTSS guidelines for complex PTSD (Cloitre et al., 2012) speaking of “exposure to repeated or prolonged instances or multiple forms of interpersonal trauma, often occurring under circumstances where escape is not possible due to physical, psychological, maturational, family/environmental, or social constraints” (p. 4). In ICD-10 (World Health Organization [WHO], 1992), complex trauma is referred to as catastrophic stress which “must be so extreme that it is not necessary to consider personal vulnerability in order to explain its profound effect on the personality” (F62.0). Many refugees, almost by definition, meet these definitions, having left their country of origin because of persecution, war, or organised violence (see www.unhcr.org).

## Complex PTSD

Several diagnoses have been put forward to describe the psychological consequences of complex trauma, most notably complex PTSD (Herman, 1992a, b), *Disorders of Extreme Stress Not Otherwise Specified* (DESNOS; Van der Kolk, Roth, Pelcovitz, Sunday, & Spinazzola, 2005), and *Enduring Personality Change After Catastrophic Experience* (EPCACE; WHO, 1992). Of these, DESNOS has been most extensively studied. DESNOS was originally intended as an independent DSM diagnosis consisting of six symptom clusters: alterations in regulation of affect and impulses, in attention or consciousness, in self-perception, in relations with others and in systems of meaning, and somatisation. However, the DSM-IV (American Psychiatric Association, 2000) field trials conducted to test the validity of the DESNOS construct did not substantiate the idea of an independent diagnosis, as only 4 to 6% of participants had DESNOS without PTSD (Van der Kolk et al., 2005). Consequently, in DSM-IV, DESNOS symptoms were described as additional characteristics of PTSD but not included in any formal diagnosis. In DSM-5, several symptoms traditionally referred to as “complex” have been incorporated into the regular PTSD diagnosis: persistent and exaggerated negative beliefs or expectations about oneself, others, or the world; persistent negative emotional state; reckless or self-destructive behaviour; and depersonalisation and derealisation (Friedman, 2013).

Existence of a clearly delineated complex posttraumatic syndrome has been more explicitly acknowledged in ICD than in DSM. ICD-10, to this date, contains the only formal diagnosis of complex PTSD, be it under a different name: Enduring Personality Change After Catastrophic Experience or EPCACE. EPCACE is characterised by a hostile or distrustful attitude towards the world, social withdrawal, feelings of emptiness or hopelessness, a chronic feeling of “being on edge” as if constantly threatened, and estrangement. Patients cannot be diagnosed as experiencing both EPCACE and PTSD. More recently, a proposal for inclusion of a complex PTSD diagnosis in ICD-11 has been formulated in which complex PTSD may be diagnosed in addition to regular PTSD. Apart from PTSD criteria, this proposal consists of disturbances in emotion regulation, a diminished and defeated sense of self, and difficulties in maintaining relationships (Maercker et al., 2013). Although ICD is the dominant diagnostic system worldwide and therefore most likely to be used in transcultural research, not EPCACE, but DESNOS has been examined in several studies with refugees, which we will describe later.

## Trauma-focused and phased treatment

The clinical relevance of recognising the existence of complex PTSD in a patient is because the diagnosis is believed to merit a treatment plan that is different from that recommended by treatment guidelines for PTSD in adults (e.g., NICE, 2005). Treatment guidelines for PTSD in adults recommend trauma-focused treatment as a first-line intervention for all patients with chronic PTSD. Trauma-focused treatment can be defined as treatment that focuses on “the patients’ memories of their traumatic events and the personal meanings of the trauma” (Ehlers et al., 2010, p. 270; Bisson et al., 2007)—examples are prolonged exposure or eye movement desensitisation and reprocessing therapy (EMDR). However, the ISTSS guidelines for complex PTSD recommend the implementation of phased treatment. This consists of a first phase that focuses on safety, symptom reduction, and skills training; a second phase that focuses on processing of traumatic memories; and a third phase that focuses on social and psychological (re-)integration. Stand-alone trauma-focused treatment is believed to carry a risk of psychologically overwhelming the patient and consequently of psychological decompensation (Herman, 1992b).

The theory of phased treatment for complex PTSD and its emphasis on safety and psychological decompensation has had a major impact on treatment for traumatised asylum seekers and refugees. For a long time, the emphasis has been on protecting refugee patients from emotional overburdening through trauma-focused therapy by delaying or avoiding trauma-focused treatment. The NICE guidelines state that:

The first need is to achieve safety from further persecution. (…) It can be hard to confront trauma memories anyway, but if the PTSD sufferer faces a realistic prospect of being returned to face more trauma, then it can be impossible. (p. 120)

Describing the second phase of treatment in a refugee suffering from complex PTSD, Momartin and Coello (2006) write:

Because of the severe and complicated nature of the present case, exposure-based treatment, as advised by Luxenberg [Luxenberg, Spinazzola, Hunt, & Van der Kolk, 2001] was not used. Instead, close attention was paid for signs or accounts of dissociation, helping the patient in ‘grounding’ himself. (p. 25)

In other words, unsafe circumstances and complex PTSD are frequently used as reasons for deviating from treatment guidelines for PTSD in refugees.

## Appraisal

In preparation for DSM-5, the central assumptions concerning complex PTSD have been extensively evaluated. On the one hand, a merit of the complex PTSD construct is that it has drawn attention to posttraumatic symptoms of survivors who are relatively out of the public eye, such as maltreated children and victims of domestic violence. This has contributed to an extension of the DSM-5 PTSD diagnosis to the 20 symptoms that it consists of now, enabling clinicians to diagnose and treat a wider range of survivors who might otherwise not be recognised as experiencing PTSD. The model of phased treatment has contributed to an examination of the possibility of harm in PTSD outcome studies, something that is now routinely recommended (Ioannidis et al., 2004). On the other hand, as shown above, little clarity has yet been reached on the relationship between PTSD and complex PTSD, with complex PTSD having been diagnostically separated from PTSD in ICD-10, added to PTSD in ICD-11, and incorporated into PTSD in DSM-5. Nor has agreement been reached on which specific traumatic events or conditions constitute a risk factor for development of complex PTSD. Finally, treatment outcome research of complex PTSD has been criticised for insufficiently distinguishing between complex traumatic events and complex posttraumatic sequelae. In-depth evaluations of the complex PTSD literature may be found in Resick et al. (2012) and Landy, Wagner, Brown-Bowers, and Monson (2015). We now turn to the two central theses of this paper.

## Theses

### I. Complex trauma leads to complex PTSD in a minority of refugees only

As noted in the introduction, there is a tendency in the complex PTSD literature to equate complex trauma and complex PTSD. Palic and Elklit (2011), for example, hypothesise that “instances of (…) complex PTSD may actually make up the largest fraction of PTSD patients when we consider the vast numbers of refugees worldwide (…)” (p. 9). Even though potentially traumatic events lead to regular PTSD in a minority of cases only, this hypothesis seems built on the assumption that all refugees who have experienced complex trauma are likely to suffer from complex PTSD.

Cloitre et al. (2009) modify this expectation by hypothesising complex PTSD to be as prevalent in refugees as in survivors of childhood trauma:

Studies of [refugee survivors of torture, political persecution, war zones, or concentration camps] might show equally powerful effects for adult and childhood cumulative trauma. Indeed, adulthood traumas of sustained nature such as living in a war zone create a life condition that increases risk of exposure to a multiplicity of types of traumatic events (e.g., actual or threat of injury, sexual assault, witnessing injury or death to others) and the accumulation of such experiences would be expected to increase risk for symptom complexity. (p. 406)

The question is whether research supports these hypotheses.

To examine this, we used studies yielded by a systematic search conducted for a meta-analysis of prevalence of complex PTSD in traumatised populations (Ter Heide, Smid, Mooren, & Kleber, in preparation). In this meta-analysis, only studies using a comprehensive instrument to assess complex PTSD were included, which is currently limited to the Structured Interview for Disorders of Extreme Stress Not Otherwise Specified (SIDES; Pelcovitz et al., 1997), which assesses DESNOS. Our search yielded five studies on prevalence of complex PTSD in refugees: three studies of treatment-seeking populations and two of non-treatment-seeking populations (one of which of refugees hosted in their own region of origin; see Table 1).

**Table 1:** Current Prevalence of Complex PTSD in Refugees

Publication	Sample	CPTSD instrument	PTSD instrument	*N*	CPTSD total, *n* (%)	PTSD total, *n* (%)	CPTSD plus PTSD, *n* (%)
De Jong et al., 2005	Eritrean refugees living in temporary shelters in Ethiopia	SIDES	CIDI	1200	26 (2.2)	190 (15.8)	Missing
Teodorescu et al., 2012	Treatment-seeking refugees resettled in Norway	SIDES	SCID-I	61	10 (16.4)	50 (82.0)	9 (14.6)
Weine et al., 1998	Bosnian refugees resettled in the US	SIDES	PSS	24	0 (0)	10 (41.7)	0 (0)
Palic & Elklit, 2014	Treatment-seeking Bosnian refugees resettled in Denmark	SIDES-SR	None	116	39 (33.6)	n.a.	n.a.
Teegen & Vogt, 2002	Treatment-seeking refugees resettled in Germany	SIDES-SR	PCL-C	33	22 (66.7)	31 (93.9)	Missing

(C)PTSD, (complex) posttraumatic stress disorder; SIDES(-SR), Structured Interview for Disorders of Extreme Stress (self-report); CIDI, International Diagnostic Interview; SCID-I, Structured Clinical Interview for DSM-IV Axis-I; PSS, PTSD Symptoms Scale; PCL-C, PTSD Checklist for DSM-IV.

Table 1 shows the different associations between complex PTSD and PTSD which we described in the introduction: some studies examine the co-occurrence of complex PTSD (DESNOS) and PTSD, while some examine the two conditions separately. The column “CPTSD total” shows the total number of participants who met a complex PTSD (DESNOS) diagnosis with or without PTSD, the column “PTSD total” shows the total number of participants who met a PTSD diagnosis with or without complex PTSD (DESNOS), and the column “CPTSD plus PTSD” shows the number of participants from the previous two columns who met both diagnoses. The first three studies provide the highest level of evidence by using the validated, clinician-rated version of the SIDES. We will look at these studies first.

First, these three studies show that refugees, both treatment-seeking and non-treatment-seeking, are much more likely to meet a PTSD diagnosis or neither diagnosis than to meet a diagnosis of complex PTSD (DESNOS). This does not necessarily mean that complex trauma does not generate complex psychosocial reactions in refugees, but that in refugees these reactions are unlikely to be captured by a complex PTSD diagnosis. In other words, the expectation that refugees in general suffer from complex PTSD, based on their experiences alone, is not supported by the data.

**Fig. 1:**
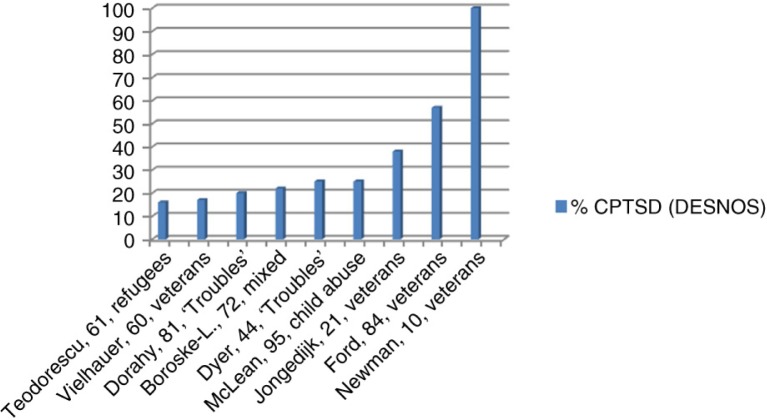
Complex PTSD in treatment-seeking samples.

Second, we used data from the first three studies to examine the hypothesis that complex PTSD may be as prevalent in refugees as in survivors of childhood traumatic experiences. Prevalence of complex PTSD after childhood trauma has only been examined in treatment-seeking samples. We therefore compared the treatment-seeking refugee sample (Teodorescu, Heir, Hauff, Wentzel-Larsen, & Lien, 2012) with other samples seeking treatment for psychotrauma-related reasons (see Fig. 1; the study descriptions state first author, sample size, and sample type). Visually, this comparison shows that total complex PTSD prevalence (i.e., with or without comorbid PTSD) is lowest in the refugee sample.

The one study addressing childhood sexual abuse is the study by McLean, Toner, Jackson, Desrocher, and Stuckless (2006), with a total complex PTSD prevalence of 25% (compared to 16% in refugees). Data obtained in the DSM-IV Field Trial (Van der Kolk et al., 2005) may also serve as a meaningful comparison. Most, but not all, of the participants in the DSM-IV Field Trial were treatment-seeking, and they were divided into three groups: survivors of early onset abuse, of late onset abuse, and of disasters. As data from this study are limited to prevalence of “complex PTSD plus PTSD,” this study was not included in Fig. 1. When compared to the DSM-IV Field Trial participants, the prevalence rate of 15% found in treatment-seeking refugees is comparable to that found in survivors of late onset abuse (18%), but lower than that found in survivors of early onset abuse (24%), and higher than that found in survivors of disasters (3%). In other words, these limited data do not confirm the hypothesis that treatment-seeking refugees are at equal risk of having complex PTSD as treatment-seeking survivors of childhood trauma.

**Fig. 2:**
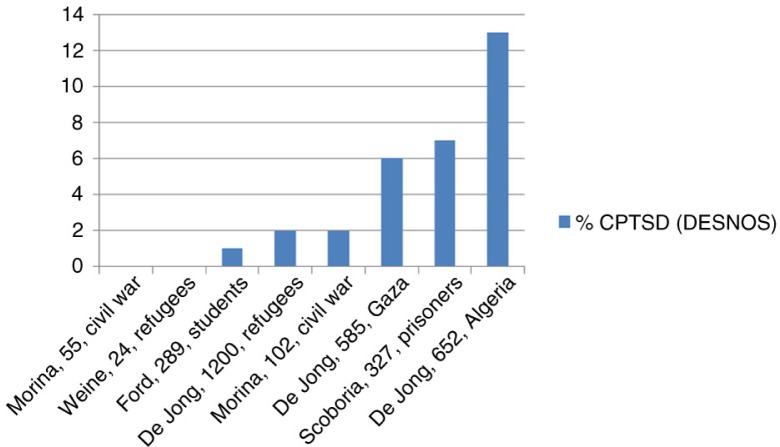
Complex PTSD in non-treatment-seeking samples.

Third, we return to the non-treatment-seeking refugee samples (De Jong, Komproe, Spinazzola, Van der Kolk, & Van Ommeren, 2005; Weine et al., 1998). To place their prevalence rates into perspective, we compare them to prevalence rates from other trauma-exposed, non-treatment-seeking samples (Fig. 2). Like the studies in Fig. 1, these rates concern prevalence of complex PTSD with or without PTSD.

Again, the refugee studies are among the studies with the lowest complex PTSD prevalence, indicating that being a refugee carries no increased risk of a complex PTSD diagnosis.

Last, two of the refugee studies in Table 1 used an unvalidated self-report version of the SIDES. Although these studies point towards a higher prevalence of complex PTSD in treatment-seeking refugees, studies using self-report only have been shown to risk overestimation of prevalence rates of up to 50% (Richardson, Frueh, & Acierno, 2010), and results would therefore need to be confirmed using a clinician-rated instrument.

In conclusion, the limited evidence that is currently available points to a complex PTSD diagnosis in only a minority of refugees. Although prevalence of complex PTSD in treatment-seeking refugees appears higher than in non-treatment-seeking refugees, it does not appear to reach as high a prevalence as in other treatment-seeking populations, including patients who have experienced childhood trauma. Clinically, this implies that complex PTSD in refugees should not be assumed to be present on the basis of traumatic experience but should be carefully diagnosed using a validated interview. In addition, there is a need for studies that use clinician-rated interviews to determine if specific refugee populations, such as former child soldiers or survivors of sexual exploitation, are at increased risk of developing complex PTSD in comparison to general refugee populations.

### II. Trauma-focused treatment should be offered to all refugees who seek treatment for PTSD

As stated earlier, phased treatment is often recommended for refugees who experience PTSD, as stand-alone trauma-focused treatment is feared to carry a risk of psychologically overwhelming refugee patients (e.g., Nickerson, Bryant, Silove, & Steel, 2011). In clinical practice, this recommendation has sometimes led to extensive or exclusive stabilisation of refugee patients. Recommendations for phase-based treatment are currently experience-based as no randomised controlled trials (RCT's) of phase-based treatment have been conducted in refugees. However, the evidence supporting the safety and efficacy of trauma-focused treatment in refugees has been accumulating.

Randomised research into the efficacy of refugee treatment has only started at the turn of the century. In the first systematic review on psychological treatment of PTSD in adult refugees (Nicholl & Thompson, 2004), only one RCT was mentioned which compared the efficacy of exposure therapy and cognitive-behaviour therapy (CBT)—both of which were found efficacious (Paunovic & Öst, 2001). Great impetus has since been provided by two research groups: one that has yielded numerous trials of narrative exposure therapy (NET; Schauer, Neuner, & Elbert, 2005), and one that has yielded several trials of culturally adapted CBT (CA-CBT; Hinton, Rivera, Hofmann, Barlow, & Otto, 2012). In recent years, numerous systematic reviews and meta-analyses have appeared which draw conclusions on treatment of traumatised refugees. Increasingly these point to the efficacy of trauma-focused treatment. In the first review of NET, Robjant and Fazel (2010) conclude that: “Emerging evidence suggests that NET is an effective treatment for PTSD in individuals who have been traumatised by conflict and organised violence, even in settings that remain volatile and insecure” (p. 1030). This statement is echoed in a systematic review by Mørkved et al. (2014).

A systematic review by Nickerson et al. (2011) looks beyond NET to the efficacy of trauma-focused interventions (NET, exposure, CBT, and CA-CBT) and multimodal treatment in refugees. Although acknowledging methodological shortcomings, they conclude that the evidence points to efficacy of trauma-focused interventions only, noting that

Most of the randomized controlled trials reviewed here reported large effect sizes in relation to PTSD symptom reduction following a trauma-focused treatment. (…) effect sizes of greater than 1.5 were common. This corresponds to a 70% or greater non-overlap of the treated group's scores with the scores at baseline. (p. 407)

Recently, the efficacy of trauma-focused treatment in refugees was subject of a meta-analysis by Lambert and Alhassoon (2015). They used the between-groups effect sizes of trauma-focused treatment (NET, CA-CBT, and EMDR) versus control groups to calculate an aggregate effect size. They found a large effect size for trauma-focused treatment in refugees, both with regards to PTSD (Hedge's *g*=0.91, *p*<0.001, 95% CI [0.56, 1.52]) and depression (Hedge's *g=*0.63, *p*<0.001, 95% CI [0.35, 0.92]). These large effect sizes are chiefly based on NET and CA-CBT, with limited evidence for EMDR (Acarturk et al., 2015; Ter Heide, Mooren, Kleijn, De Jongh, & Kleber, 2011; Ter Heide, Mooren, Knipscheer, & Kleber, 2014). In summary, contrary to suggestions in the complex PTSD literature current evidence supports the efficacy of trauma-focused treatment, especially NET and CA-CBT, in refugee samples.

The need for stabilisation is thought to most strongly apply to refugees who live in unstable social settings, such as asylum seekers, whose refugee claim is still under consideration, and refugees who are hosted within their own region of origin rather than in western resettlement countries. Although this social instability really constitutes a different kind of complexity than that captured in the complex PTSD construct, the two kinds of complexity are often equated, as in the ISTSS guidelines for complex PTSD. What is the evidence for trauma-focused treatment in these groups? The meta-analysis by Lambert and Alhassoon contains five studies that included refugees in unstable settings: two of NET with Sudanese, Rwandan, and Somalian refugees hosted in Uganda (Neuner et al., 2008; Neuner, Schauer, Klaschik, Karunakara, & Elbert, 2004); and two of NET (Neuner et al., 2010; Stenmark, Catani, Neuner, Elbert, & Holen, 2013) and one of EMDR (Ter Heide et al., 2011) with asylum seekers hosted in Western Europe. All NET studies showed large effect sizes for PTSD symptom reduction from pretreatment to follow-up assessment (Hedge's *g* of 1.6, 1.4, and 1.6 for regional refugees in Neuner et al., 2004, 2008, and 2010, respectively; and Hedge's *g* of 0.93 for asylum seekers in Stenmark et al., 2013). In the EMDR study, which does not give effect sizes for asylum seekers separately, it was stated that asylum seekers and those with a refugee status had an equal chance of dropping out of treatment. In other words: there is currently no evidence that shows that refugees in unstable settings and asylum seekers are unable to benefit from trauma-focused treatment and that with these groups, trauma-focused treatment should therefore be avoided or extensively delayed (see also De Jong, Knipscheer, Ford, & Kleber, 2014).

On the basis of these findings, it may be concluded that there is no scientific justification for the clinical practice of extensively or solely stabilising refugees. There is no randomised research on the efficacy of phase-based treatment for refugees with PTSD. There is accumulating evidence that supports the efficacy of trauma-focused treatment in refugees. Although not all refugees may wish to undergo trauma-focused treatment (e.g., Morris et al., 1993) and not all refugees may benefit equally (Schottenbauer, Glass, Arnkoff, Tendick, & Gray, 2008), there is no indication that complex PTSD symptoms predict refusal, dropout, or non-response to trauma-focused treatment in refugees and that extensive stabilisation is therefore necessary. Those refugees who are reluctant to participate in primarily exposure-based treatment such as NET may benefit from CA-CBT which provides a combination of skills training and exposure. Whether phased treatment in refugees leads to greater acceptability, lower dropout, and higher efficacy than trauma-focused treatment *per se* is a matter of great interest. However, with the current state of research it is more accurate to recommend a course of trauma-focused treatment for all refugees seeking treatment for PTSD, including asylum seekers, than to recommend psychosocial stabilisation as a prerequisite for trauma-focused treatment.

## Conclusion

All clinicians and researchers working with refugees, regardless of whether or not they endorse the concepts of complex PTSD and phased treatment in refugees, strive to alleviate suffering in this highly burdened group of patients. What this paper aims at is not so much polarising the debate as encouraging careful diagnostics of traumatised refugees while discouraging the practice of long-term stabilisation in refugees who are perceived as too vulnerable for trauma-processing. Although there may be valid clinical reasons to postpone trauma-focused treatment in refugees, such as untreated psychosis, we pose that there is currently no evidence that justifies the use of complex PTSD and lack of refugee status as exclusion criteria for trauma-focused treatment in refugees in general. This paper is an invitation to further debate and we look forward to any response that promotes helpful diagnostics and treatment for traumatised refugees.
